# Twelve Weeks of Yoga or Nutritional Advice for Centrally Obese Adult Females

**DOI:** 10.3389/fendo.2018.00466

**Published:** 2018-08-17

**Authors:** Shirley Telles, Sachin K. Sharma, Niranjan Kala, Sushma Pal, Ram K. Gupta, Acharya Balkrishna

**Affiliations:** Patanjali Research Foundation, Haridwar, India

**Keywords:** central obesity, anthropometry, anthropometric indices, risk of disease, yoga, nutritional advice

## Abstract

**Background:** Central obesity is associated with a higher risk of disease. Previously yoga reduced the BMI and waist circumference (WC) in persons with obesity. Additional anthropometric measures and indices predict the risk of developing diseases associated with central obesity. Hence the present study aimed to assess the effects of 12 weeks of yoga or nutritional advice on these measures. The secondary aim was to determine the changes in quality of life (QoL) given the importance of psychological factors in obesity.

**Material and Methods:** Twenty-six adult females with central obesity in a yoga group (YOG) were compared with 26 adult females in a nutritional advice group (NAG). Yoga was practiced for 75 min/day, 3 days/week and included postures, breathing practices and guided relaxation. The NAG had one 45 min presentation/week on nutrition. Assessments were at baseline and 12 weeks. Data were analyzed with repeated measures ANOVA and *post-hoc* comparisons. Age-wise comparisons were with *t*-tests.

**Results:** At baseline and 12 weeks NAG had higher triglycerides and VLDL than YOG. Other comparisons are within the two groups. After 12 weeks NAG showed a significant decrease in WC, hip circumference (HC), abdominal volume index (AVI), body roundness index (BRI), a significant increase in total cholesterol and LDL cholesterol. YOG had a significant decrease in WC, sagittal abdominal diameter, HC, BMI, WC/HC, a body shape index, conicity index, AVI, BRI, HDL cholesterol, and improved QoL. With age-wise analyses, in the 30–45 years age range the YOG showed most of the changes mentioned above whereas NAG showed no changes. In contrast for the 46–59 years age range most of the changes in the two groups were comparable.

**Conclusions:** Yoga and nutritional advice with a diet plan can reduce anthropometric measures associated with diseases related to central obesity, with more changes in the YOG. This was greater for the 30–45 year age range, where the NAG showed no change; while changes were comparable for the two groups in the 46–59 year age range. Hence yoga may be especially useful for adult females with central obesity between 30 and 45 years of age.

Trial registration: (CTRI/2018/05/014077).

## Background

The Indian Council of Medical Research-India Diabetes (ICMR-INDIAB) study is an ongoing cross sectional national study of the prevalence of obesity, diabetes, and hypertension across the Indian sub-continent (1). In Phase 1 of the study 16,000 individuals aged 20 years and above were sampled from the whole of India. Generalized, abdominal, and combined obesity (i.e., generalized as well as abdominal) were determined based on the BMI and waist circumference using the World Health Organization Asia Pacific Guidelines (2, 3). The ICMR-INDIAB study reported a higher prevalence of isolated abdominal obesity compared to generalized obesity across India. This finding was given importance considering the association independent of ethnicity, between central obesity and several chronic non-communicable diseases such as cardiovascular disease, type 2 diabetes mellitus and certain cancers (1).

Lifestyle changes which include increased physical activity, a healthy diet and a positive attitude have been recommended for the management of obesity (4). For people who are obese and physically less active, physical activity may seem challenging (5). This would prevent persons who are obese from initiating and adhering to increased physical activity. The practice of yoga has been adopted for weight control because of its increasing popularity and relative safety while supervised (6, 7). A systematic meta-analysis assessed the effects of yoga for weight related outcomes in overweight and obese persons assessed in randomized controlled trials (RCTs) (7). Thirty trials with a total of 2,173 participants from bibliographic databases such as MEDLINE, Scopus, and Cochrane Library were screened from their inception to March 2016. The risk of bias was assessed using the Cochrane risk of bias tool which demonstrated methodological drawbacks in the 30 trials. Nonetheless the authors of the meta-analysis concluded that yoga can be considered a safe and effective intervention to reduce the BMI in overweight and obese individuals.

A single study demonstrated a decrease in central obesity in 60 female participants after 12 weeks of yoga (8). This inference was based on the waist circumference. There are several other anthropometric measures and derived anthropometric indices which have been shown to predict the risk of developing diseases associated with central obesity (9).

The aim of this study was to determine if in adult females with central obesity a 12 week program of yoga or of nutritional advice could (i) alter anthropometric measurements associated with a risk of developing diseases associated with central obesity and (ii) positively influence the lipid profile and quality of life.

## Materials and methods

### Participants

**Figure 1:**
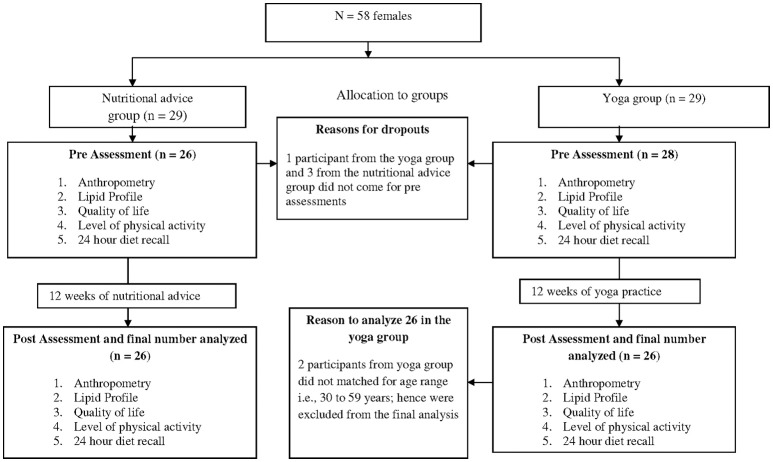
Trial profile of the study.

Fifty two healthy Asian Indian adult females with central obesity with ages between 30 and 59 years (group average age ± SD; 43.98 ± 6.89 years) were recruited for the trial. The trial profile is provided in Figure 1. The sample size was determined from a previous study on centrally obese females (8). A required sample size (*n* = 20) was obtained using Cohen's formula for the effect size of 0.40 calculated from the mean and SD values of waist circumference which were changed significantly after 12 weeks of yoga, alpha at 0.05, powered at 0.90 using G power software (10). Participants were recruited through advertisements in local newspapers and flyers distributed in nearby residential areas and hospitals. Participation in the study was voluntary with no remuneration. The inclusion criteria were (i) waist circumference ≥ 80 cm (11), (ii) BMI ≥ 25 kg/m^2^ (12), (iii) ages between 30 and 59 years, and (iv) willingness to take part in the study. The exclusion criteria were (i) obesity secondary to hormonal imbalance, medication such as steroids or secondary to any other medical condition, (ii) any physical or psychological disability which would have prevented the participants from taking part in the yoga program or attending the nutritional advice session, (iii) involvement in any other dietary or exercise program during the 12 months prior to, at the time of or during the study, and (iv) any co-morbidities associated with obesity such as cardiovascular disease, type-2 diabetes mellitus, or hypertension. No participant was excluded from the trial for the above mentioned reasons. The participants' written signed informed consent was taken. The baseline characteristics of the participants are given in Table 1.

**Table 1:** The baseline characteristics of the participants.

**Characteristics**	**Nutritional advice group**	**Yoga group**
Number of participants (n)	26	26
Number of participants in 30–45 (years) age group	12	16
Number of participants in 46–59 (years) age group	14	10
Age in years (mean ± SD)	45.9 ± 7.4	42.5 ± 8.3
Weight in kg (mean ± SD)	74.62 ± 12.03	78.92 ± 12.03
BMI in kg/m^2^ (mean ± SD)	31.75 ± 4.17	33.31 ± 4.71
Waist circumference in cm (mean ± SD)	100.77 ± 10.79	101.06 ± 8.56
Type of diet	Vegetarian	Vegetarian
Health	Normal	Normal
Taking any medication	No	No
Consumption of alcohol or nicotine in any form	No	No

### Study design

The present single blind comparative controlled trial was carried out between April and August 2016 where assessors were blinded to the group to which the participants belonged. The yoga group practiced yoga for 75 min/day, for 3 consecutive days in a week, over a 12 week period. Along with this they were given a diet plan for 1,900–2,000 Kcal/day developed based on the guidelines from the National Institute of Nutrition, India (13). The nutritional advice group was given lectures on nutrition (one 45 min lecture/week) and the same diet plan for 1,900–2,000 Kcal/day developed based on the guidelines from the National Institute of Nutrition, India (13). Adherence to yoga was based on the attendance in the yoga class noted by the yoga teacher. The compliance to the diet plan was based on the 24 h diet recall questionnaire which was administered at the start and end of the 12 week period. This did not cover the dietary intake during the rest of the time which is a limitation. Both groups were assessed for levels of physical activity using the International Physical Activity Questionnaire—Short Form administered at the beginning and end of 12 weeks. The study had the approval of the ethics committee of the institute, which was formed and based on the guidelines of the Indian Council of Medical Research and is in accordance with the Helsinki Declaration (Approval number: YRD/016/022). The trial is registered with the Clinical Trials Registry of India (CTRI/2018/05/014077). The present study is part of a larger nationwide trial of which the data are still being analyzed, comparing yoga with nutritional advice in different regions of India.

At the start of the trial an attempt was made to convince participants to be randomly allocated to yoga or nutritional advice groups. However participants had time and practical constraints. Hence based on their convenience they were assigned to either intervention. However several participants of the nutritional advice group mentioned that they were interested to learn yoga later on.

### Assessments

The following assessments were carried out by individuals who were blinded to the group to which the participants belonged.

### Anthropometry

#### Waist circumference (WC)

Participants were lightly clothed and asked to stand upright with their feet 25–30 cm apart and weight evenly distributed on both feet. The tape measure which was used for assessments (Gülick Anthropometric tape Model J00305, Lafayette Instrument, U.S.A.) was fitted around the abdomen without compressing soft tissue. The waist circumference was measured to the nearest 0.1 cm in a horizontal plane midway between the inferior costal margin and the iliac crest.

#### Sagittal abdominal diameter (SAD)

The participant was asked to lie supine on their back. The caliper used to measure the supine sagittal abdominal diameter has two arms attached to a vertical scale [Holtain-Kahn Abdominal Caliper 50 cm (98.609XL), U.K.]. The standard method was followed by which the lower arm of the caliper was placed under the participant (14). After a normal exhalation, the upper arm of the caliper was lowered to reach the mid-point between the inferior costal margin and the iliac crest. The lower arm was re-positioned if required. The reading on the vertical scale between the upper and lower arms of the caliper gave the supine sagittal abdominal diameter in cm.

#### Hip circumference (HC)

The hip circumference was measured around the pelvis at the point of maximal protrusion of the buttocks. The ratio of the waist circumference to the hip circumference was derived and is a ratio of the fat stored centrally inside the abdomen (waist circumference) and fat stored peripherally (hip circumference).

#### Derived anthropometric indices

Seven anthropometric indices were derived from direct measurements using standardized formulae (9, 15–18). These indices are as follows.

#### Body mass index (BMI)

The body mass index (BMI) was calculated as the body weight (in kg), in light clothing and without footwear, divided by the height (in meters squared). The accuracy of the weighing machine (Model DS 215 N, Essae-Teraoka Pvt. Ltd, Bengaluru, India) was up to 0.05 kg. The height was measured to the nearest 0.1 cm.

BMI = Weight (kg)/ Height ^2^(m).

#### Waist-hip ratio (WHR)

WHR = WC (cm)/HC (cm).

#### A body shape index (ABSI) (9)

ABSI = WC (m)/[BMI ^2/3^ (kg/m^2^) Height ½ (m)]

where WC and height are expressed in m and BMI in kg/m^2^.

#### Conicity index (CI) (9)

CI = 0.109 ^−1^ WC (m) [Weight (kg) / Height (m)] ^−1/2^

where WC is measured in cm, weight in kg and height in m.

#### Abdominal volume index (AVI) (9)

AVI = [2WC^2^ (cm) + 0.7 (WC - HC) ^2^ (cm)]/1000

where WC and HC (hip circumference) are expressed in cm.

#### Visceral adiposity index (VAI) (15)

VAI _female_ = {WC (cm)/[36.58 +1.89BMI (kg/m^2^)]} [TG (mmol/L)/0.81] [1.52/HDL (mmol/L)]

where WC is expressed in cm, BMI in kg/m^2^, Triglycerides in mmol/L, and HDL in mmol/L.

#### Body roundness index (BRI) (9)

BRI = 364.2 – 365.5 [1 –π ^−2^ WC ^2^ (m) Height ^−2^ (m)] ^1/2^

where WC and height are expressed in m.

### Biochemical measures

Antecubital venous blood samples were collected under sterile conditions. Total cholesterol, triglycerides, high density, and low density lipoprotein cholesterol were estimated using appropriate enzymes followed by spectrophotometry.

### 24 h diet recall (energy intake/day)

A structured interview was carried out to recall the food and fluids which the participants had consumed in the 24 h prior to the study. The method was as follows: participants were asked (i) to recall and list the foods they had consumed during the 24 h preceding the assessment, (ii) the method of preparation (e.g., raw, cooked, boiled, or baked), (iii) the size of utensils used, the interviewer had four types of utensils (i.e., a bowl, cup, glass, and spoon), each of which had four sizes. Participants were asked to indicate the size used by them. The volume of each of the utensils of different sizes had already been determined (19). The total energy intake/day as well as the amount of macronutrients was calculated based on norms for Indian foods (13, 19, 20).

### Level of physical activity and energy expenditure/day

Physical activity was assessed using the International Physical Activity Questionnaire Short Form (IPAQ) (21). Data obtained at the beginning and end of the 12 week period for both groups were extracted using the guidelines published by the IPAQ for data processing (22). From these data the basal metabolic rate of each participant was determined and energy expenditure/day was calculated using the Harris-Benedict equation (23).

### Quality of life

The Moorehead-Ardelt Quality of Life Questionnaire was used to assess six aspects of the quality of life (QoL) (24). These were general self-esteem, physical activity, social contacts, satisfaction concerning work, pleasure related to sexuality, and focus on eating behavior, with scores ranging from −0.5 to +0.5. The sum of these 6 scores provided a total QoL score. Each score was classified into 5 categories (very poor: −3.0 to −2.1; poor: −2.0 to −1.1; fair: −1.0 to +1.0; good: 1.1 to 2.0: and very good: 2.1 to 3.0).

### Interventions

#### Nutritional advice

Participants received a 45 min presentation on nutrition (1 presentation/week) for 12 weeks. The person who gave the presentation had a minimum of 12 years of education including 2 years of training in science. The presenter used slides made by the research institute conducting the trial. The 12 topics of the presentations were: (i) five basic food groups, (ii) vegetarian diet, (iii) proteins, (iv) fats, (v) carbohydrates, (vi) dietary fiber, (vii) vitamins, (viii) minerals, (ix) probiotics, (x) iron deficiency, (xi) calcium, and (xii) antioxidants.

#### Yoga

The yoga intervention consisted of (i) a universal prayer (3 min)^1^, (ii) yoga posture*s (asanas*, 42 min), (iii) voluntarily regulated breathing techniques (*pranayamas*, 24 min), and (iv) guided relaxation with meditation (6 min). Yoga classes were conducted on three successive days in a week between (i) 05:30 h and 06:45 h, during the 12 weeks. Attendance was noted in each yoga class by the yoga instructor. The instructor was also asked to note any adverse event during the classes. The yoga instructor had been teaching yoga for 5 years. All the participants in the yoga group attended at least 18 out of a total of 36 yoga classes. Details of the yoga intervention are given in Table 2.

**Table 2:** Details of the yoga intervention.

**SI. no**.		**Name of the yoga practice**	**Duration of the practice**
1	Universal prayer	*Sukhasana* (easy posture) + *Prathana mantra* (universal prayer)	3 min
2	*Pranayama* (voluntarily regulated yoga breathing)	1. *Bhastrika* (bellows yoga breathing)	3 min
		2. *Kapalabhati* (high frequency yoga breathing)	6 min
		3. *Ujjayi* (victorious breathing)	3 min
		4. *Anulom-vilom* (alternate nostril yoga breathing)	6 min
		5. *Bhramari* (bumble bee breathing)	3 min
		6. *Udgeeth* (OM chanting)	3 min
3	*Asanas* Standing postures	1. *Tirryaktadasana* (swaying palm tree pose) (right side, left side with 10 repetitions; normal breathing and eyes closed)	3 min
		2. *Trikonasana* (triangle pose) (right side followed by left side; the posture is sustained for at least 15 s on either side with eyes closed and normal breathing)	3 min
		3. *Konasana* (angle pose) (right side followed by left side; the posture is sustained for at least 15 s on either side with eyes closed and normal breathing)	3 min
		4. *Padahastasana* (hand to foot pose) (remaining in the posture for at least 15 s with normal breathing and eyes closed)	1 min
4	*Asanas* Sitting postures	1.*Chakkiasana* (mill churning pose) seated with legs extended, hands stretched out and fingers interlaced. Each movement would involve forward bending and making circles with the extended arms keeping the spine erect (15 rounds, clockwise; 15 rounds anti-clockwise with normal breathing and eyes closed)	3 min
		2. *Sthitta konaasana* (static angle pose) Extending both legs and keeping them one forearm span apart while seated, then holding the left big toe with the right hand while the left arm is extended upward. The person should face the extended left arm keeping the spine erect. During the practice there should be normal breathing and eyes closed. The posture should be maintained for at least 15 s. The practice is repeated on the opposite side.	3 min
		3. *Paschimottanasana* (seated forward bend pose) (remaining in the posture for at least 15 s with normal breathing and eyes closed)	1 min
5	*Asanas* Supine postures	1. *Ardhahalasana* (half plow pose) raising both legs to form a right angle, keeping the legs straight at the knee, repetitive (10 times with eyes closed and normal breathing)	3 min
		2. *Padavrttasana* (cyclical leg pose) raising the right leg extended at the knee and making circles in the air, rotating the leg (10 rounds clockwise and 10 rounds anticlockwise with normal breathing and eyes closed). The practice is repeated with the left leg with 10 rounds clockwise and 10 rounds anticlockwise.	6 min
		3. *Dwicakriasana* (cycling pose) repetitive (10 times with eyes closed and normal breathing)	3 min
		4. *Markatasana* (monkey pose) this is a spinal twist (right side, left side with 10 repetitions; normal breathing and eyes closed)	6 min
6	*Asanas* Prone postures	*1. Bhujangaasana* (cobra pose) two methods are followed. Method1—Lie prone with extended elbows and the palms near the chest, flat on the ground. Then the upper part of the body till the waist is raised while looking upwards and forwards. The weight of the upper parts of the body should be evenly distributed on both hands. The procedure is repeated 10 times with eyes closed and normal breathing. The second method is almost the same except that the hands are not kept apart but with the right palm over the left palm. The rest of the procedure is the same. This method is also repeated 10 times with normal breathing and eyes closed.	6 min
		2. *Salabhasana* (locust pose) (remaining in the posture for at least 15 s with normal breathing and eyes closed)	1 min
7	Guided relaxation	*Shavasana* (corpse pose) with breath awareness	6 min
		**Total duration**	75 min

*Those practices which are commonly used are not detailed*.

#### Diet plan

Participants of both groups were given a diet plan for a balanced diet of 1900–2000 Kcal/day. The diet plan included fruits, vegetables, lentils, complex carbohydrates, and dairy products. Hence it was a lacto-vegetarian diet. The ratio of carbohydrates, protein and fats in the diet was based on the guidelines for a balanced diet for an Indian population published by the National Institute of Nutrition, India (13), which states that a balanced Indian diet should have 10–15% of total calories from proteins, 20–30% of calories from fats and 50–60% of calories from carbohydrates. The total energy and nutritive values of the foods were determined from a database of Indian foods (20).

1. ^1^May all be prosperous and happy
2. May all be free from illness
3. May all see what is uplifting
4. May no one suffer
5. Peace, peace, peace

### Data analysis

#### Group as a whole

The data obtained at the beginning and end of the 12 week period for the two groups were compared with a repeated measures analysis of variance (ANOVA) followed by multiple *post-hoc* comparisons which were Bonferroni adjusted. The ANOVA had one Within subjects factor i.e., States, with two levels (pre and post) and one Between subjects factor i.e., Groups which were (i) nutritional advice group with a diet plan of 1900–2000 Kcal/day and (ii) yoga group who had a similar diet plan of 1900–2000 Kcal/day, as the nutritional advice group.

#### Analysis based on age

Both nutritional advice and yoga groups were subdivided based on age as (i) participants between 30 and 45 years and (ii) those who were 46 years and above.

For (i) and (ii) there were separate between group comparisons of values at baseline and after 12 weeks using *t*-tests for unpaired data. Within the groups baseline data and date at 12 weeks were compared with *t*-tests for paired data.

## Results

None of the participants reported any adverse events during the trial. At baseline the visceral adiposity index (VAI), levels of triglycerides and of VLDL differed significantly between the nutritional advice and yoga groups. The group mean values ± SD for the (i) anthropometric measures and anthropometric indices, (ii) lipid profile, (iii) energy intake/day and energy expenditure/day, and (iv) quality of life are given in Tables 3–7.

**Table 3:** Anthropometric variables.

**Variables**	**Nutritional advice (*****n*** **=** **26)**					**Yoga (*****n*** **=** **26)**				
	**Pre**	**Post**	**Cohen's d**	**95% CI**		**Pre**	**Post**	**Cohen's d**	**95% CI**	
				**Lower**	**Upper**				**Lower**	**Upper**
Waist circumference (cm)	100.8 ± 10.8	97.9 ± 8.6^*^	0.30	0.72	5.00	101.1 ± 8.6	94.8 ± 7.1^**^	0.81	4.14	8.41
SAD (cm)	23.5 ± 2.8	23.3 ± 2.3	0.08	−0.35	0.73	24.1 ± 2.6	23.1 ± 2.6^**^	0.39	0.45	1.52
Hip circumference (cm)	111.7 ± 7.4	109.2 ± 6.8^**^	0.36	0.79	4.16	112.3 ± 10.4	107.8 ± 9.3^***^	0.46	2.79	6.16
BMI (kg/m^2^)	31.8 ± 4.2	31.3 ± 4.1	0.12	−0.10	1.06	33.3 ± 4.7	32.2 ± 5.4^***^	0.22	0.52	1.68
Waist/hip ratio	0.9 ± 0.1	0.9 ± 0.1	0.17	−0.01	0.03	0.9 ± 0.1	0.9 ± 0.1^*^	0.31	0.00	0.04
A body shape index	0.081 ± 0.01	0.079 ± 0.01^@^	0.22	−0.02	0.29	0.079 ± 0.01	0.06 ± 0.01^*^	0.21	0.04	0.35
Conicity index	1.3 ± 0.1	1.3 ± 0.1	0.21	−0.00	0.05	1.3 ± 0.2	1.3 ± 0.1^**^	0.28	0.01	0.06
Abdominal volume index	20.7 ± 4.3	19.4 ± 3.4^**^	0.33	0.44	2.02	21.2 ± 6.1	19.3 ± 5.6^***^	0.33	1.08	2.65
Visceral adiposity index	2.9 ± 1.4^#^	3.0 ± 2.1	0.02	−0.51	0.44	2.2 ± 1.1	2.3 ± 0.9	0.07	−0.51	0.37
Body roundness index	6.9 ± 2.0	6.4 ± 1.5^**^	0.30	0.18	0.84	7.0 ± 2.4	6.3 ± 2.2^***^	0.33	0.40	1.06

*Values are group mean± SD*.

* p < 0.05;

** p < 0.01;

*** *p < 0.001, post-hoc analyses with Bonferroni adjustment, post values compared with pre*.

# *p < 0.05, post-hoc analyses with Bonferroni adjustment, pre values compared with pre*.

@ *p < 0.05, post-hoc analyses with Bonferroni adjustment, post values compared with post*.

*BMI, Body mass index; SAD, Sagittal abdominal diameter*.

**Table 4:** Lipid profile.

**Variables**	**Nutritional advice (*****n*** = **26)**					**Yoga (*****n*** = **26)**				
	**Pre**	**Post**	**Cohen's d**	**95% CI**		**Pre**	**Post**	**Cohen's d**	**95% CI**	
				**Lower**	**Upper**				**Lower**	**Upper**
Total cholesterol (mmol/L)	4.6 ± 0.8	5.0 ± 0.8^**^	0.54	−0.7	−0.15	4.6 ± 0.8	4.6 ± 1.0	0.02	−0.3	0.23
Triglycerides (mmol/L)	1.72 ± 0.7^#^	1.7 ± 0.7^@@^	0.01	−0.21	0.19	1.35 ± 0.5	1.30 ± 0.42	0.18	−0.11	0.27
LDL cholesterol (mmol/L)	2.8 ± 0.6	3.0 ± 0.6^*^	0.41	−0.44	−0.02	3.1 ± 0.6	3.0 ± 0.7	0.15	−0.08	0.27
HDL cholesterol(mmol/L)	1.2 ± 0.3	1.3 ± 0.3	0.13	−0.14	0.06	1.23 ± 0.24	1.1 ± 0.3^*^	0.50	0.03	0.22
VLDL (mmol/L)	0.77 ± 0.3^#^	0.78 ± 0.29^@@^	0.04	−0.10	0.08	0.58 ± 0.24	0.56 ± 0.15	0.12	−0.06	0.11

Values are group mean ± SD.

* p < 0.05;

** p < 0.01, post-hoc analyses with Bonferroni adjustment, post values compared with pre.

@@ p < 0.01, post-hoc analyses with Bonferroni adjustment, post values compared with post.

# *p < 0.05, post-hoc analyses with Bonferroni adjustment, pre values compared with pre*.

**Table 5:** Estimated energy intake/day based on 24 h diet recall questionnaire.

**Variables**	**Nutritional advice (*****n*** = **26)**					**Yoga (*****n*** = **26)**				
	**Pre**	**Post**	**Cohen's d**	**95% CI**		**Pre**	**Post**	**Cohen's d**	**95% CI**	
				**Lower**	**Upper**				**Lower**	**Upper**
Protein (gm)/day	53.8 ± 17.4	55.7 ± 10.3	0.13	−9.15	5.44	59.0 ± 14.0	50.7 ± 10.4	0.68	1.24	15.23
Fat (gm)/day	47.5 ± 19.6	46.9 ± 18.5	0.03	−8.87	10.03	39.2 ± 14.1	41.4 ± 16.7	0.15	−11.88	7.39
Carbohydrates (gm)/day	193.6 ± 76.9	222.0 ± 77.5	0.38	−79.07	22.12	216.5 ± 55.4	248.7 ± 114.2	0.37	−82.78	18.42
Energy intake (Kcal)/day	1625.9 ± 395.4	1716.3 ± 384.6	0.24	−320.57	139.89	1753.5 ± 423.5	1590.6 ± 366.0	0.42	−67.33	393.13

*Values are group mean ± SD*.

**Table 6:** Estimated energy expenditure/day based on (i) International Physical Activity Questionnaire—Short Form and (ii) Harris-Benedict equation to determine the basal metabolic rate.

**Variable**	**Nutritional advice (*****n*** = **26)**					**Yoga (*****n*** = **26)**				
	**Pre**	**Post**	**Cohen's d**	**95% CI**		**Pre**	**Post**	**Cohen's d**	**95% CI**	
				**Lower**	**Upper**				**Lower**	**Upper**
Total energy (Kcal) spent/day	2022.6 ± 238.7	1995.6 ± 205.7	0.12	−87.79	141.78	2158.8 ± 322.6	2019.5 ± 219.8	0.52	24.10	253.67

*Values are group mean ± SD*.

**Table 7:** Quality of life.

**Variables**	**Nutritional advice (*****n*** = **26)**					**Yoga (*****n*** = **26)**				
	**Pre**	**Post**	**Cohen's d**	**95% CI**		**Pre**	**Post**	**Cohen's d**	**95% CI**	
				**Lower**	**Upper**				**Lower**	**Upper**
General self-esteem	0.22 ± 0.27	0.3 ± 0.16	0.37	−0.185	0.015	0.22 ± 0.25	0.30 ± 0.16	0.39	−0.188	0.012
Physical activity	0.25 ± 0.23	0.32 ± 0.15	0.37	−0.165	0.026	0.24 ± 0.23	0.25 ± 0.22	0.05	−0.130	0.61
Social contacts	0.22 ± 0.29	0.30 ± 0.15	0.35	−0.104	0.081	0.24 ± 0.23	0.30 ± 0.17	0.30	−0.130	0.168
Satisfaction concerning work	0.32 ± 0.14	0.35 ± 0.12	0.24	−0.108	0.046	0.29 ± 0.25	0.29 ± 0.22	0.00	−0.085	0.069
Pleasure related to sexuality	0.04 ± 0.31	0.14 ± 0.31	0.33	−0.196	0.034	0.07 ± 0.31	0.14 ± 0.25	0.25	−0.184	0.046
Focus on eating behavior	0.29 ± 0.22	0.27 ± 0.21	0.09	−0.072	0.110	0.2 ± 0.3	0.29 ± 1.9^*^	0.07	−0.183	−0.001
Total quality of life	1.34 ± 0.93	1.67 ± 0.78	0.39	−0.675	0.014	1.23 ± 1.06	1.58 ± 0.87^*^	0.37	−0.690	−0.002

Values are group mean ± SD.

* *p < 0.05, post-hoc analyses with Bonferroni adjustment, post values compared with pre*.

### Repeated measures analysis of variance (RM-ANOVA)

The ANOVA values for the Within-Subjects factor (States), Between-Subjects factor (Groups) and interaction between the two are given below. The details of the ANOVA are available in the Supplementary Material.

### Within-subjects factor (states)

There were significant differences between post and pre states for waist circumference (*F* = 36.92, *p* < 0.001), sagittal abdominal diameter (*F* = 9.66, *p* < 0.01), hip circumference (*F* = 34.41, *p* < 0.001), BMI (*F* = 14.88, *p* < 0.001), a body shape index (*F* = 20.77, *p* < 0.01), conicity index (*F* = 13.09, *p* < 0.001), abdominal volume index (*F* = 31.05, *p* < 0.001), body roundness index (*F* = 28.78, *p* < 0.001), total cholesterol (*F* = 5.71, *p* < 0.05), general self-esteem (*F* = 6.05, *p* < 0.05), and total quality of life (*F* = 7.80, *p* < 0.01). For all comparisons mentioned above the condition of sphericity was met and value of epsilon (Huynh-Feldt, Greenhouse-Geisser) was 1. The degrees of freedom for all variables were 1,50.

### Between-subjects factors (groups)

There were significant differences between the groups in a body shape index (*F* = 4.10, *p* < 0.05), triglycerides (*F* = 8.23, *p* < 0.01), and VLDL (*F* = 9.94, *p* < 0.01). The condition of sphericity was met for the comparisons and value of epsilon (Huynh-Feldt, Greenhouse- Geisser) was 1. The degrees of freedom for all variables were 1,50.

### Interaction between states and groups

Interaction between States and Groups was significant for waist circumference (*F* = 5.11, *p* < 0.05), total cholesterol (*F* = 4.69, *p* < 0.05), LDL cholesterol (*F* = 5.77, *p* < 0.05), and HDL cholesterol (*F* = 5.44, *p* < 0.05). A significant interaction between States and Groups suggests that the effects of the two factors are interdependent. For all comparisons the condition of sphericity was met and value of epsilon (Huynh-Feldt, Greenhouse-Geisser) was 1. The degrees of freedom for all variables were 1, 50 (States) x 50 (Groups).

### *Post-hoc* analyses

There were two *post-hoc* comparisons: (i) Between groups [at baseline (pre) and at 12 weeks (post)] and (ii) Within groups, comparing values obtained at 12 weeks (post) with those at baseline (pre).

### *Post-hoc* between groups comparisons

At baseline (pre) the visceral adiposity index (VAI, *p* < 0.05), VLDL (*p* < 0.05) and triglyceride levels (*p* < 0.05) were significantly higher in the nutritional advice group compared to the yoga group. After 12 weeks (post) a body shape index (*p* < 0.01), the triglyceride levels (*p* < 0.01), and VLDL (*p* < 0.01) levels were higher in the nutritional advice group compared to the yoga group.

### *Post-hoc* comparisons within a group (post-pre)

The nutritional advice group showed a significant decrease in the waist circumference (*p* < 0.05), hip circumference (*p* < 0.01), abdominal volume index (*p* < 0.01), body roundness index (*p* < 0.01), a significant increase in total cholesterol (*p* < 0.01), and LDL cholesterol (*p* < 0.05).

The yoga group had a significant decrease in waist circumference (*p* < 0.001), sagittal abdominal diameter (*p* < 0.01), hip circumference (*p* < 0.001), BMI (*p* < 0.001), waist/hip ratio (*p* < 0.05), a body shape index (*p* < 0.05), conicity index (*p* < 0.01), abdominal volume index (*p* < 0.001), body roundness index (*p* < 0.001), HDL cholesterol (*p* < 0.05), improved quality of life, i.e., focus on eating behavior (*p* < 0.05) and total quality of life (*p* < 0.05).

### Age wise analysis (*t*-tests)

The participants of both groups were divided as two groups based on age, viz., 30–45 years and 46–59 years. For each age group, comparisons were made between nutritional advice and yoga groups using *t*-tests for unpaired data and within each group using *t*-tests for paired data. The age wise group mean values ± SD for the (i) anthropometric measures and anthropometric indices, (ii) lipid profile, (iii) energy intake/day and energy expenditure/day, and (iv) quality of life are given in Tables 8–12.

**Table 8:** Anthropometric variables and derived indices for two age range i.e., 30-45 and 46-59 year.

**Variables**	**Nutritional advice**				**Yoga**			
	**Pre**	**Post**	***t* value**	**Cohen's d**	**Pre**	**Post**	***t* value**	**Cohen's d**
**Age range**	**30–45 Years age range**							
Number of participants (n)	12				16			
Waist circumference (cm)	97.3 ± 12.3	98.0 ± 10.6	0.52	0.07	101.6 ± 9.3	95.3 ± 8.4^***^	5.77	0.71
SAD (cm)	22.0 ± 2.7	22.6 ± 2.5	1.25	0.25	24.0 ± 2.6	23.1 ± 2.8^**^	3.34	0.34
Hip circumference (cm)	110.8 ± 9.5	109.7 ± 9.0	0.70	0.12	113.2 ± 11.8	108.9 ± 9.8^**^	4.25	0.40
BMI (kg/m^2^)	30.5 ± 4.8	30.6 ± 4.5	0.20	0.02	33.1 ± 4.9	32.0 ± 4.7^**^	4.08	0.24
Waist/hip ratio	0.9 ± 0.1	0.9 ± 0.1	0.88	0.17	0.9 ± 0.1	0.9 ± 0.1	1.98	0.25
A body shape index	0.1 ± 0.0	0.1 ± 0.0	0.368	0.00	0.08 ± 0.0	0.08 ± 0.0^**^	4.0	0.31
Conicity index	1.3 ± 0.1	1.3 ± 0.1	0.75	0.10	1.3 ± 0.2	1.3 ± 0.2^*^	2.68	0.18
Abdominal volume index	19.4 ± 4.9	19.5 ± 4.2	0.31	0.04	22.3 ± 7.3	20.5 ± 6.7^***^	4.48	0.27
Visceral adiposity index	2.9 ± 1.4	2.6 ± 1.3	0.92	0.26	2.1 ± 1.0	2.3 ± 0.8	1.65	0.28
Body roundness index	6.2 ± 2.1	6.3 ± 1.8	0.35	0.04	7.4 ± 2.9	6.7 ± 2.6^**^	4.01	0.26
**Age range**	**46–59 Years**							
Number of participants (n)	14				10			
Waist circumference (cm)	103.8 ± 8.7^#^	97.9 ± 6.8^***^	3.26	0.79	100.2 ± 7.7	93.9 ± 4.8^**^	3.33	1.03
SAD (cm)	24.8 ± 2.3	23.9 ± 2.0^**^	4.64	0.42	24.2 ± 2.7	23.1 ± 2.3^*^	2.99	0.46
Hip circumference (cm)	112.5 ± 5.2	108.8 ± 4.4^**^^@^	3.30	0.80	110.8 ± 8.1	106.2 ± 8.7^**^	4.59	0.59
BMI (kg/m^2^)	32.9 ± 3.4	31.9 ± 3.7	2.15	0.28	33.6 ± 4.6	32.6 ± 4.1^*^	2.36	0.24
Waist/hip ratio	0.9 ± 0.1	0.9 ± 0.1^*^	2.19	0.32	0.9 ± 0.0	0.9 ± 0.0	1.07	0.30
A body shape index	0.08 ± 0.0	0.08 ± 0.0^**^	3.43	0.10	0.08 ± 0.0	0.1 ± 0.0^*^	2.94	0.42
Conicity index	1.4 ± 0.1	1.3 ± 0.1^**^	4.18	0.77	1.3 ± 0.1	1.2 ± 0.1	2.01	0.55
Abdominal volume index	21.8 ± 3.5	19.4 ± 2.6^***^	4.61	0.82	19.4 ± 2.7	17.4 ± 2.2^**^	3.30	0.82
Visceral adiposity index	2.9 ± 1.4	3.3 ± 2.6	0.79	0.20	2.4 ± 1.2	2.2 ± 1.0	1.11	0.19

*Values are group mean ± SD*.

* p < 0.05;

** p < 0.01;

*** *p < 0.001, paired t-test, post values compared with pre*.

@ *p < 0.05, Unpaired t-test, post values compared with post*.

# *p < 0.05, Unpaired t-test, pre values compared with pre*.

*BMI, Body mass index; SAD, Sagittal abdominal diameter*.

**Table 9:** Lipid profile for two age range i.e., 30–45 and 46–59 year.

**Variables**	**Nutritional advice**				**Yoga**			
	**Pre**	**Post**	***t* value**	**Cohen's d**	**Pre**	**Post**	***t* value**	**Cohen's d**
**Age range**	**(30–45 Years)**							
Number of participants (n)	12				16			
Total cholesterol (mmol/L)	4.58 ± 0.78	5.43 ± 0.62	3.71	1.26	4.34 ± 0.77	4.20 ± 0.74	1.59	0.19
Triglycerides (mmol/L)	1.75 ± 0.80^#^	1.64 ± 0.52	0.55	0.17	1.22 ± 0.36	1.24 ± 0.34	0.39	0.07
LDL cholesterol (mmol/L)	2.95 ± 0.49	3.41 ± 0.48	3.37	0.98	2.89 ± 0.59	2.76 ± 0.62	1.72	0.22
HDL cholesterol (mmol/L)	1.17 ± 0.19	1.33 ± 0.24	1.60	0.74	1.22 ± 0.22	1.08 ± 0.20	3.33	0.68
VLDL (mmol/L)	0.75 ± 0.32	0.72 ± 0.20	0.39	0.13	0.54 ± 0.17	0.54 ± 0.13	0.20	0.05
**Age range**	**(46–59 Years)**							
Number of participants (n)	14				10			
Total cholesterol (mmol/L)	4.55 ± 0.84	4.54 ± 0.78	0.04	0.01	5.08 ± 0.55	5.35 ± 0.86	1.14	0.39
Triglycerides (mmol/L)	1.70 ± 0.56	1.82 ± 0.79	0.78	0.19	1.56 ± 0.62	1.32 ± 0.42	1.59	0.48
LDL cholesterol (mmol/L)	2.65 ± 0.60	2.62 ± 0.43	0.19	0.05	3.36 ± 0.42	3.31 ± 0.83	0.25	0.07
HDL cholesterol (mmol/L)	1.28 ± 0.40	1.20 ± 0.32	1.09	0.23	1.28 ± 0.28	1.18 ± 0.35	1.78	0.33
VLDL (mmol/L)	0.78 ± 0.26	0.84 ± 0.38	0.78	0.19	0.66 ± 0.31	0.60 ± 0.19	0.88	0.25

Values are group mean ± SD.

# *p < 0.05, Unpaired t-test, pre values compared with pre*.

**Table 10:** Estimated energy intake/day based on 24 h diet recall questionnaire for two different age range i.e., 30–45 and 46–59 year.

**Variables**	**Nutritional advice**				**Yoga**			
	**Pre**	**Post**	***t* value**	**Cohen's d**	**Pre**	**Post**	***t* value**	**Cohen's d**
**Age range**	**(30–45 Years)**							
Number of participants (n)	12				16			
Protein (gm)/day	51.9 ± 19.3	55.9 ± 12.0	0.54	0.26	64.5 ± 13.5	53.6 ± 8.9	2.79	0.98
Fat (gm)/day	52.3 ± 22.1	54.9 ± 17.5	0.34	0.17	37.8 ± 13.2	42.7 ± 17.8	0.77	0.32
Carbohydrates (gm)/day	198.0 ± 76.9	216.8 ± 60.8	0.68	0.28	238.2 ± 45.5	251.1 ± 97.6	0.43	0.18
Energy intake (Kcal)/day	1601.4 ± 447.1	1738.8 ± 292.3	0.83	0.38	1744.6 ± 299.8	1679.9 ± 425.1	0.49	0.18
**Age range**	**(46–59 Years)**							
Number of participants (n)	14				10			
Protein (gm)/day	55.9 ± 15.7	55.5 ± 8.6	0.09	0.03	50.7 ± 9.9	46.4 ± 11.5	1.03	0.42
Fat (gm)/day	43.4 ± 16.9	40.1 ± 16.9	0.53	0.20	41.7 ± 16.0	39.2 ± 15.3	0.37	0.17
Carbohydrates (gm)/day	189.8 ± 79.5	226.5 ± 91.5	1.09	0.45	181.8 ± 53.8	244.7 ± 142.6	1.12	0.62
Energy intake (Kcal)/day	1646.9 ± 361.3	1696.9 ± 459.6	0.29	0.13	1767.8 ± 589.9	1447.7 ± 184.8	1.58	0.77

*Values are group mean ± SD*.

**Table 11:** Estimated energy expenditure/day based on (i) International Physical Activity Questionnaire—Short Form and (ii) Harris-Benedict equation to determine the basal metabolic rate for two different age range i.e., 30-45 and 46-59 years.

**Variables**	**Nutritional advice (*****n*** = **12)**				**Yoga (*****n*** = **16)**			
	**Pre**	**Post**	***t* value**	**Cohen's d**	**Pre**	**Post**	***t* value**	**Cohen's d**
**Age range**	**(30–45 Years)**							
Number of participants (n)	12				16			
Total energy (Kcal) spent/day	2027.8 ± 275.2	2035.0 ± 233.8	0.07	0.03	2173.0 ± 299.5	2007.1 ± 226.9	2.40	0.65
**Age range**	**(46–59 Years)**							
Number of participants (n)	14				10			
Total energy (Kcal) spent/day	2018.2 ± 213.2	1961.9 ± 180.0	0.75	0.30	2136.2 ± 372.5	2040.6 ± 218.3	1.00	0.33

*Values are group mean ± SD*.

**Table 12:** Quality of life for two different age range i.e., 30–45 and 46–59 years.

**Variables**	**Nutritional advice**				**Yoga**			
	**Pre**	**Post**	***t* value**	**Cohen's d**	**Pre**	**Post**	***t* value**	**Cohen's d**
**Age range**	**(30–45 Years)**							
Number of participants (n)	12				16			
General self-esteem	0.3 ± 0.2	0.29 ± 0.19	0.15	0.06	0.2 ± 0.3	0.3 ± 0.2	0.99	0.28
Physical activity	0.3 ± 0.2	0.3 ± 0.2	0.24	0.06	0.3 ± 0.2	0.3 ± 0.2	0.11	0.00
Social contacts	0.2 ± 0.3	0.3 ± 0.2	1.27	0.60	0.2 ± 0.3	0.3 ± 0.2	0.63	0.23
Satisfaction concerning work	0.3 ± 0.2	0.3 ± 0.1	0.0	0.00	0.4 ± 0.2	0.3 ± 0.2	0.48	0.17
Pleasure related to sexuality	0.1 ± 0.3	0.1 ± 0.3	0.14	0.04	0.2 ± 0.2	0.2 ± 0.2	0.89	0.30
Focus on eating behavior	0.3 ± 0.2^#^	0.2 ± 0.2	0.89	0.33	0.2 ± 0.3	0.3 ± 0.2	1.23	0.44
Total quality of life	1.4 ± 0.7	1.5 ± 0.9	0.36	0.09	1.5 ± 0.9	1.8 ± 0.8	1.15	0.32
**Age range**	**(46-59 Years)**							
Number of participants (n)	14				10			
General self-esteem	0.2 ± 0.4	0.3 ± 0.1	1.93	0.58	0.2 ± 0.3	0.3 ± 0.1	1.59	0.65
Physical activity	0.2 ± 0.3	0.4 ± 0.1	1.97	0.74	0.1 ± 0.3	0.2 ± 0.2	1.27	0.34
Social contacts	0.3 ± 0.3	0.3 ± 0.2	0.60	0.17	0.3 ± 0.2	0.2 ± 0.2	0.16	0.05
Satisfaction concerning work	0.3 ± 0.1^#^	0.4 ± 0.1^@^	1.37	0.72	0.2 ± 0.3	0.2 ± 0.3	1.21	0.25
Pleasure related to sexuality	−0.0 ± 0.3	0.1 ± 0.3	1.58	0.44	−0.1 ± 0.4	0.0 ± 0.3	0.85	0.29
Focus on eating behavior	0.3 ± 0.3	0.3 ± 0.2	0.38	0.10	0.2 ± 0.3	0.3 ± 0.1	1.72	0.63
Total quality of life	1.3 ± 1.1	1.8 ± 0.7	1.87	0.62	0.8 ± 1.2	1.3 ± 1.0^*^	2.57	0.47

Values are group mean±SD.

* p < 0.05, Paired t-test, post values compared with pre.

@ p < 0.05, Unpaired t-test, post values compared with post.

# *p < 0.05, Unpaired t-test, pre values compared with pre*.

### Age group 30–45 years

#### Between groups

At baseline the triglyceride levels and the focus on eating behavior (an aspect of the quality of life) were lower in the yoga group (*p* < 0.05, both cases) compared to the nutritional advice group. There were no differences at 12 weeks between groups.

#### Within groups

There were no significant changes in the nutritional advice group. In the yoga group there was a decrease in waist circumference, sagittal abdominal diameter, hip circumference, BMI, a body shape index, conicity index, abdominal volume index, and body roundness index (*p* < 0.05 in all cases).

### Age group 46–59 years

#### Between groups

At baseline the waist circumference and satisfaction concerning work (of the quality of life scale) were higher in the nutritional advice group compared to the yoga group (*p* < 0.05). At 12 weeks the hip circumference and satisfaction concerning work remained higher in the nutritional advice group compared to the yoga group (*p* < 0.05 both cases).

#### Within groups

The nutritional advice group showed a decrease in waist circumference, sagittal abdominal diameter, hip circumference, waist-hip ratio, a body shape index, conicity index, abdominal volume index, and body roundness index (*p* < 0.05 in all cases). The yoga group had a significant decrease in waist circumference, sagittal abdominal diameter, hip circumference, BMI, a body shape index, abdominal volume index, body roundness index, and total quality of life (*p* < 0.05 in all cases). The significant results have been summarized in the Supplementary Material.

## Discussion

Following 12 weeks of nutritional advice there was a significant decrease in waist circumference, hip circumference, abdominal volume index, and increase in total cholesterol and LDL cholesterol. The yoga group at the end of 12 weeks showed a decrease in waist circumference, sagittal abdominal diameter, hip circumference, BMI, waist-hip ratio, a body shape index, conicity index, abdominal volume index, body roundness index, HDL cholesterol, and better total quality of life. When both groups were considered as two age ranges (i.e., 30–45 and 46–59 years), the results were different. For the 30–45 years age group the nutritional advice group showed no change after 12 weeks whereas the yoga group showed most of the changes mentioned above for the group as a whole. In contrast to this for the 46–59 year age group, the nutritional advice and yoga groups showed comparable benefits with reduction in most-anthropometric measures and indices at 12 weeks. Hence yoga may be especially useful for adult females between 30 and 45 years of age.

The waist circumference, hip circumference, abdominal volume index, and body roundness decreased in both groups irrespective of age. The waist circumference correlates with increased risk of cardiovascular disease (25), the abdominal volume index has been correlated with impaired glucose tolerance and higher incidence of type 2 DM and metabolic syndrome (26). Body roundness index was associated with higher occurrence of non-alcoholic fatty liver disease (27). Hence the risk of these conditions could be considered to be lower in both groups at 12 weeks. The other changes in the yoga group also suggest a reduction in the risk of developing obesity associated diseases such as carotid artery stiffness (28) and insulin resistance (29), based on a decrease in SAD, cardiovascular disease (based on reduction in waist-hip ratio and conicity index (30), and cardiometabolic disease (based on reduced a body shape index and BMI) (31). Hence these risk factors reduced in the yoga group irrespective of age.

The serum lipid profile was assessed using quantitative methods. The yoga group showed a significant decrease in HDL cholesterol. This reduction in HDL cholesterol has been seen in two other studies, in which obese participants received yoga for 6 days and 15 days (32, 33). In both studies participants consumed a plant based lacto vegetarian diet comparable to that of the present study. In another study, 12 weeks of yoga practice resulted in a significant decrease in total cholesterol, triglycerides and LDL levels with a non-significant increase in HDL levels (34). The participants' diet was not described. It has been observed that those diets which are most effective in reducing the risk of atherosclerosis are usually associated with the greatest decrease in protective HDL cholesterol levels (35–37). These diets are typically plant based high fiber low fat diets. However this reduction in protective HDL cholesterol levels following such diets need not necessarily be harmful as separate studies have shown that even if HDL levels decrease, the anti-inflammatory efficacy of HDL cholesterol may be enhanced despite reduction in absolute levels of HDL (38, 39).

In the nutritional advice group there was a significant increase in total cholesterol, and LDL levels. The explanation for this increase is not clear as the nutritional advice group was given the same dietary instructions as the yoga group. Mental stress levels have a positive correlation with total cholesterol, triglycerides and LDL cholesterol levels (40–42). Practicing yoga is one of the methods for stress reduction. Even though the stress levels were not measured in the present study it may be speculated that in the absence of yoga intervention the nutritional advice group continued to experience higher stress levels which contributed to the increase in total cholesterol, and LDL cholesterol levels. Though this is a speculation it is supported by the results of the participants' response to the six items of the Moorehead-Ardelt Quality of Life Questionnaire.

The yoga group showed significantly higher scores in focus on eating behavior (an aspect of quality of life) and total quality of life after 12 weeks. Previously the Moorehead-Ardelt Quality of Life Questionnaire has been used to compare the quality of life in obese persons who were experienced in yoga compared to those without any yoga experience and demonstrated a better quality of life in the group with prior yoga experience (43). These findings are of importance as psychological wellbeing is important for the long term successful management of obesity (44).

The centrally obese participants of the present trial showed no differences in their energy intake or energy expenditure after 12 weeks irrespective of the group to which they belonged. Energy intake was derived from the 24 h diet recall questionnaire which does not give an accurate idea of the diet during the 12 week period. Hence though the present results suggest that the energy intake in a day did not differ significantly with the energy expenditure in a day, between groups after 12 weeks it must be emphasized that both energy intake/day and energy expenditure/day were assessed by qualitative methods which lack the accuracy and objectivity of quantitative assessments.

The main limitations of the present findings are the study design and small sample size. Both the yoga and nutritional advice groups were given their intervention based on convenience, though the nutritional advice group did express an interest to learn yoga at some stage after the trial. The ideal design would have been a randomized controlled trial but after recruitment it was clear that though the participants were motivated to learn yoga, for personal reasons such as time constraints they were unable to state that they could complete 12 weeks of yoga practice successfully. This point demonstrates the practical difficulties a person may have in learning and practicing any intervention. Apart from this, though the supine sagittal abdominal diameter through anthropometry is an acceptable method to measure visceral adipose tissue, magnetic resonance imaging (MRI), dual-energy x-ray absorptiometry (DEXA), and computed tomography (CT) scans would be more accurate (30). To distinguish between types of adipose tissue in central obesity these methods are essential. The other limitations include the lack of quantitative measures to assess energy expenditure/day and energy intake/day. Also the present sample included females alone, all of whom were generational vegetarians. Hence generalizing the findings cannot be done.

## Conclusions

Yoga and nutritional advice with a diet plan can reduce anthropometric measures associated with diseases related to central obesity, with more changes in the yoga group. This difference was greater for the 30–45 years age range, where the nutritional advice group showed no change; while changes were comparable for the two groups in the 46–59 year age range. Hence yoga may be especially useful for adult females with central obesity between 30 and 45 years of age.

## Author contribution

ST, SS, and AB designed the study. NK and SP performed data collection and analyses. ST and SS wrote the manuscript. NK, SP, and RG prepared the manuscript. ST, SS, NK, SP, RG, and AB proofread the manuscript.

### Conflict of interest statement

The authors declare that the research was conducted in the absence of any commercial or financial relationships that could be construed as a potential conflict of interest. The reviewer OH and handling Editor declared their shared affiliation.
