# Supplementary material for: Twelve Weeks of Yoga or Nutritional Advice for Centrally Obese Adult Females
Source: Front Endocrinol (Lausanne). 2018 Aug 17;9:466. doi: 10.3389/fendo.2018.00466 (PMC6107686; doi:10.3389/fendo.2018.00466)
Supplement: Supplementary file 1 [file Table_1.DOC]

**Supplementary table 1:** ANOVA table for the anthropometric variables.

| **SI. no.** | **Factors** | **Variable** | ***F*** | ***df*** | **Huynh-Feldt ε** | ***p* value** |
| --- | --- | --- | --- | --- | --- | --- |
| I | Within Subjects | BMI | 14.88 | 1,50 | 1 | <0.001 |
|  |  | Hip circumference | 34.41 | 1,50 | 1 | <0.001 |
|  |  | Waist circumference | 36.92 | 1,50 | 1 | <0.001 |
|  |  | Waist/hip ratio | 3.69 | 1,50 | 1 | 0.060 |
|  |  | Sagittal abdominal diameter | 9.66 | 1,50 | 1 | 0.003 |
|  |  | A body shape index | 9.52 | 1,50 | 1 | 0.003 |
|  |  | Conicity index | 13.09 | 1,50 | 1 | 0.001 |
|  |  | Abdominal volume index | 31.05 | 1,50 | 1 | <0.001 |
|  |  | Visceral adiposity index | 0.10 | 1,50 | 1 | 0.751 |
|  |  | Body roundness index | 28.78 | 1,50 | 1 | <0.001 |
| II | Between Subjects | BMI | 1.08 | 1,50 | 1 | 0.305 |
|  |  | Hip circumference | 0.03 | 1,50 | 1 | 0.875 |
|  |  | Waist circumference | 0.37 | 1,50 | 1 | 0.546 |
|  |  | Waist/hip ratio | 0.16 | 1,50 | 1 | 0.691 |
|  |  | Sagittal abdominal diameter | 0.05 | 1,50 | 1 | 0.827 |
|  |  | A body shape index | 0.13 | 1,50 | 1 | 0.715 |
|  |  | Conicity index | 0.54 | 1,50 | 1 | 0.468 |
|  |  | Abdominal volume index | 0.02 | 1,50 | 1 | 0.878 |
|  |  | Visceral adiposity index | 3.91 | 1,50 | 1 | 0.054 |
|  |  | Body roundness index | 0.01 | 1,50 | 1 | 0.938 |
| III | States × Groups | BMI | 2.32 | 1,50 (States)**×** 50 (Groups) | 1 | 0.134 |
|  |  | Hip circumference | 2.85 | 1,50 (States)**×** 50 (Groups) | 1 | 0.098 |
|  |  | Waist circumference | 5.19 | 1,50 (States)**×** 50 (Groups) | 1 | 0.027 |
|  |  | Waist/hip ratio | 1.34 | 1,50 (States)**×** 50 (Groups) | 1 | 0.252 |
|  |  | Sagittal abdominal diameter | 4.45 | 1,50 (States)**×** 50 (Groups) | 1 | 0.040 |
|  |  | A body shape index | 0.30 | 1,50 (States)**×** 50 (Groups) | 1 | 0.586 |
|  |  | Conicity index | 0.75 | 1,50 (States)**×** 50 (Groups) | 1 | 0.392 |
|  |  | Abdominal volume index | 1.31 | 1,50 (States)**×** 50 (Groups) | 1 | 0.257 |
|  |  | Visceral adiposity index | 0.01 | 1,50 (States)**×** 50 (Groups) | 1 | 0.909 |
|  |  | Body roundness index | 0.90 | 1,50 (States)**×** 50 (Groups) | 1 | 0.347 |

**Supplementary table 2: ANOVA table for lipid profile.**

| **SI. no.** | **Factors** | **Variable** | ***F*** | ***df*** | **Huynh-Feldt ε** | ***p* value** |
| --- | --- | --- | --- | --- | --- | --- |
| I | Within Subjects | Total cholesterol | 5.71 | 1,50 | 1 | 0.021 |
|  |  | Triglycerides | 0.27 | 1,50 | 1 | 0.609 |
|  |  | LDL cholesterol | 0.95 | 1,50 | 1 | 0.336 |
|  |  | HDL cholesterol | 1.56 | 1,50 | 1 | 0.218 |
|  |  | VLDL cholesterol | 0.04 | 1,50 | 1 | 0.846 |
| II | Between Subjects | Total cholesterol | 0.39 | 1,50 | 1 | 0.534 |
|  |  | Triglycerides | 8.23 | 1,50 | 1 | 0.006 |
|  |  | LDL cholesterol | 0.26 | 1,50 | 1 | 0.611 |
|  |  | HDL cholesterol | 0.93 | 1,50 | 1 | 0.341 |
|  |  | VLDL cholesterol | 9.94 | 1,50 | 1 | 0.003 |
| III | States × Groups | Total cholesterol | 4.69 | 1,50 (States) x 50 (Groups) | 1 | 0.036 |
|  |  | Triglycerides | 0.39 | 1,50 (States) x 50 (Groups) | 1 | 0.533 |
|  |  | LDL cholesterol | 5.77 | 1, 50 (States) x 50 (Groups) | 1 | 0.021 |
|  |  | HDL cholesterol | 5.44 | 1, 50 (States) x 50 (Groups) | 1 | 0.024 |
|  |  | VLDL cholesterol | 0.36 | 1, 50 (States) x 50 (Groups) | 1 | 0.554 |

**Supplementary table 3:** ANOVA table for quality of life.

| **SI. no.** | **Factors** | **Variable** | ***F*** | ***df*** | **Huynh-Feldt ε** | ***p* value** |
| --- | --- | --- | --- | --- | --- | --- |
| I | Within Subjects | General self-esteem | 6.05 | 1,50 | 1 | 0.017 |
|  |  | Physical activity | 2.38 | 1,50 | 1 | 0.129 |
|  |  | Social contacts | 3.69 | 1,50 | 1 | 0.069 |
|  |  | Satisfaction concerning work | 0.50 | 1,50 | 1 | 0.482 |
|  |  | Pleasure related to sexuality | 3.44 | 1,50 | 1 | 0.070 |
|  |  | Focus on eating behavior | 1.30 | 1,50 | 1 | 0.260 |
|  |  | Total quality of life | 7.80 | 1,50 | 1 | 0.007 |
| II | Between Subjects | General self-esteem | <0.01 | 1,50 | 1 | 0.969 |
|  |  | Physical activity | 1.18 | 1,50 | 1 | 0.283 |
|  |  | Social contacts | <0.01 | 1,50 | 1 | 0.939 |
|  |  | Satisfaction concerning work | 1.15 | 1,50 | 1 | 0.289 |
|  |  | Pleasure related to sexuality | 0.21 | 1,50 | 1 | 0.648 |
|  |  | Focus on eating behavior | 0.40 | 1,50 | 1 | 0.529 |
|  |  | Total quality of life | 0.202 | 1,50 | 1 | 0.655 |
| III | States × Groups | General self-esteem | <0.01 | 1,50 (States)**×** 50 (Groups) | 1 | 0.957 |
|  |  | Physical activity | 0.27 | 1,50 (States)**×** 50 (Groups) | 1 | 0.609 |
|  |  | Social contacts | 0.182 | 1,50 (States)**×** 50 (Groups) | 1 | 0.671 |
|  |  | Satisfaction concerning work | 0.18 | 1,50 (States)**×** 50 (Groups) | 1 | 0.672 |
|  |  | Pleasure related to sexuality | 0.02 | 1,50 (States)**×** 50 (Groups) | 1 | 0.887 |
|  |  | Focus on eating behavior | 3.02 | 1,50 (States)**×** 50 (Groups) | 1 | 0.088 |
|  |  | Total quality of life | <0.01 | 1,50 (States)**×** 50 (Groups) | 1 | 0.950 |
