# Supplementary material for: Twelve Weeks of Yoga or Nutritional Advice for Centrally Obese Adult Females
Source: Front Endocrinol (Lausanne). 2018 Aug 17;9:466. doi: 10.3389/fendo.2018.00466 (PMC6107686; doi:10.3389/fendo.2018.00466)
Supplement: Supplementary file 2 [file Table_2.DOC]

| Post *hoc* analyses for the whole group | | t-tests for age group 30-45 years | | t-tests for age group 46-59 years | |
| --- | --- | --- | --- | --- | --- |
| Between group differences | Within group differences | Between group differences | Within group differences | Between group differences | Within group differences |
| Pre-Pre  1. VAI (higher in NA)  2. TG (higher in NA)  3. VLDL (higher in NA)  Post-Post  1. ABSI (higher in NA)  2.TG (higher in NA)  3. VLDL (higher in NA) | NA group  1. WC ↓  2. HC ↓  3. AVI ↓  4. BRI ↓  5. Cholesterol ↑  6. LDL ↑  Yoga group  1. WC ↓  2. SAD ↓  3. HC↓  4. BMI ↓  5. WHR ↓  6. ABSI ↓  7. CI ↓  8. AVI ↓  9. BRI ↓  10. HDL↓  11. Focus on eating behavior ↑  12. QoL total ↑ | Pre-Pre  1. TG (higher in NA)  2. Focus on eating behavior ((higher in NA)  Post-Post  Nil | NA group  Nil  Yoga group  1. WC ↓  2. SAD ↓  3. HC ↓  4. BMI ↓  5. ABSI ↓  6.CI ↓  7. AVI ↓  8. BRI ↓ | Pre-Pre  1. WC (higher in NA)  2. Satisfaction concerning work (higher in NA )  Post-Post  1. HC (higher in NA)  2. Satisfaction concerning work (higher in NA) | NA group  1. WC ↓  2. HC ↓  3. WHR ↓  4. SAD ↓  5. ABSI ↓  6. CI ↓  7. AVI ↓  8. BRI ↓  Yoga group  1.WC ↓  2. HC ↓  3. SAD ↓  4. BMI ↓  5. ABSI ↓  6. AVI ↓  7. BRI ↓  8. QoL total ↑ |

**Supplementary table 4: Summary of the results**

**Abbreviations -**  ABSI : A body shape index, AVI: Abdominal volume index, BMI: Body mass index, BRI: Body roundness index, CI: Conicity index, HDL: high density lipoprotein, HP: Hip circumference, LDL: Low density lipoprotein, NA: Nutritional advice , QoL: Quality of life, SAD: Sagittal abdominal diameter, TG: Triglycerides, VAI: Visceral adiposity index, VLDL: Very low density lipoprotein, WC: Waist circumference, WHR: Waist hip ratio.

**Supplementary table 5: Percentage change in (i) whole group, (ii) age range 30-45 years and (iii) 46-59 years in both nutritional advice and yoga group.**

| Variables | % change after nutritional advice | | | % change after yoga | | |
| --- | --- | --- | --- | --- | --- | --- |
|  | Whole group | Age 30-45 | Age 46-59 | Whole group | Age 30-45 | Age 46-59 |
| Waist circumference (cm) | -2.83* | 0.77 | -5.73*** | -6.21*** | -6.19*** | -6.26** |
| SAD (cm) | -0.81 | 2.87 | -3.47** | -4.11** | -3.79** | -4.55* |
| Hip circumference (cm) | -2.22** | -0.94 | -3.29** | -3.99*** | -3.83** | -4.23** |
| BMI (kg/m^2^) | -1.48 | 0.30 | -2.92 | -3.30*** | -3.47** | -2.97* |
| Waist/hip ratio | -1.11* | 1.14 | -2.17* | -2.22* | -2.22 | -1.11 |
| A body shape index | -2.47 | 0.00 | -3.29** | -24.05* | -3.80** | -5.13* |
| Conicity index | -1.52 | 0.77 | -4.44** | -3.08** | -2.24* | -3.97 |
| Abdominal volume index | -5.95** | 0.88 | -11.16*** | -8.78*** | -8.19*** | -9.92** |
| Visceral adiposity index | 1.37 | -11.56 | 14.09 | 3.20 | 11.54 | -8.47 |
| Body roundness index | -7.50** | 1.29 | -13.36** | -10.44*** | -9.45** | -12.30* |
| Total cholesterol (mg/dl) | 9.28** | 18.49 | -0.10 | 0.45 | -3.09 | 5.29 |
| Triglycerides (mg/dl) | 0.45 | -6.18 | 7.24 | -5.73 | 1.97 | -15.36 |
| LDL cholesterol (mg/dl) | 8.11* | 15.37 | -0.98 | -3.14 | -4.43 | -1.39 |
| HDL cholesterol (mg/dl) | 3.08 | 13.23 | -6.26 | -10.02* | -11.57 | -7.69 |
| VLDL (mg/dl) | 1.59 | -4.26 | 7.23 | -4.12 | 1.31 | -8.98 |
| General self-esteem | 36.36 | 3.57 | 93.75 | 36.36 | 26.09 | 68.42 |
| Physical activity | 28.00 | -3.45 | 71.43 | 4.17 | 0.00 | 80.00 |
| Social contacts | 36.36 | 77.78 | 15.38 | 25.00 | 20.83 | -4.00 |
| Satisfaction concerning work | 9.38 | 0.00 | 18.18 | 0.00 | -8.11 | 46.67 |
| Pleasure related to sexuality | 250.00 | 10.00 | 700.00 | 100.00 | 35.29 | 112.50 |
| Focus on eating behavior | -6.90 | -26.92 | 6.25 | 45.00* | 42.11 | 57.14 |
| Total quality of life | 24.63 | 4.90 | 43.65 | 28.46* | 17.45 | 58.54* |
